# Emerging Photoacoustic Imaging Techniques for Peripheral Arterial Disease

**DOI:** 10.1007/s11936-025-01113-2

**Published:** 2025-08-28

**Authors:** Yide Zhang, Lihong V. Wang

**Affiliations:** 1 Department of Electrical, Computer, and Energy Engineering, University of Colorado Boulder, 425 UCB, 1111 Engineering Dr ECEE 1B55, Boulder, CO 80309 USA; 2 Biomedical Engineering Program, University of Colorado Boulder, Boulder, CO 80309 USA; 3 Caltech Optical Imaging Laboratory, Andrew and Peggy Cherng Department of Medical Engineering, Department of Electrical Engineering, California Institute of Technology, 1200 East California Boulevard, Pasadena, CA 91125 USA

**Keywords:** Photoacoustic imaging, Peripheral arterial disease, Hemodynamics, Vascular imaging, Microcirculation, Translational research

## Abstract

**Purpose of Review:**

Photoacoustic imaging (PAI) has emerged as a promising non-ionizing modality that leverages optical absorption contrast to provide both anatomical and functional insights into vascular health. This review examines recent advances in PAI technologies applied to the diagnosis, assessment, and management of peripheral arterial disease (PAD). The goal is to evaluate how emerging PAI techniques address current diagnostic limitations and to identify opportunities for clinical integration.

**Recent Findings:**

Recent studies have demonstrated the potential of PAI to capture high-resolution, dynamic images of peripheral vasculature, quantify oxygen saturation and regional blood volume, and assess microvascular health. Technological innovations, including single-shot volumetric imaging, all-optical scanners, and multimodal systems, have expanded PAI’s clinical utility.

**Summary:**

Emerging PAI systems show promise for complementing traditional imaging by providing functional insights into microvascular health. Continued technological development and validation through large-scale studies are essential for establishing PAI’s clinical role in PAD diagnosis and management.

## Opinion Statement

Peripheral arterial disease (PAD) represents a substantial clinical challenge, characterized by progressive atherosclerotic narrowing of the lower extremity arteries, leading to ischemia and functional impairment. While standard treatments like lifestyle changes, medications, and revascularization are the main approaches for managing PAD, the integration of emerging photoacoustic imaging (PAI) technologies could substantially enhance diagnosis, risk stratification, and treatment planning. PAI’s ability to produce high-resolution, real-time images of both surface and small blood vessels is a key strength, especially for assessing tissue oxygen levels, blood flow, and the development of new vessels. These functional insights can complement standard anatomical imaging, like computed tomography angiography (CTA) and magnetic resonance angiography (MRA), which are essential for planning procedures but often fall short in evaluating small vessel health. A hybrid diagnostic approach that includes PAI could help evaluate vascular responses and microcirculatory health, improving how patients are selected for treatments like endovascular revascularization or supervised exercise therapy. Additionally, PAI could be used to monitor treatment success, allowing for early detection of restenosis or poor blood flow after interventions. Identifying patients at higher risk of ulceration or amputation earlier in the disease course would support timely, targeted care and potentially reduce complications. Integrating PAI into routine clinical practice offers the potential to transform PAD management by providing a thorough, noninvasive assessment of both large and small vessels, enabling more precise and personalized treatment decisions.

## Introduction

PAD is a prevalent and progressive vascular condition that affects an estimated 8–12 million people in the United States and over 200 million globally, with a growing burden in aging populations [1–3]. It is characterized by the atherosclerotic narrowing and occlusion of peripheral arteries, most commonly involving the lower extremities. PAD shares common risk factors with coronary artery disease, including smoking, diabetes, hypertension, and hyperlipidemia. Clinically, PAD often manifests as intermittent claudication, i.e., pain or cramping in the legs during exertion, and can progress to critical limb ischemia, characterized by rest pain, ulceration, and risk of limb loss [4, 5]. Beyond the local manifestations, PAD is a systemic marker of atherosclerosis, conferring a significantly increased risk of cardiovascular events such as myocardial infarction and stroke [6, 7]. Early and accurate diagnosis is essential for risk stratification, therapeutic planning, and the prevention of major adverse cardiovascular and limb events [3]. However, achieving this remains challenging due to the frequently asymptomatic nature of the disease and the limitations inherent in current diagnostic tools [8, 9].

A range of imaging modalities has been developed to aid in the diagnosis and management of PAD, each with unique technical characteristics, advantages, and limitations (Table 1) [10]. Computed tomography angiography (CTA) offers high spatial resolution and rapid acquisition, enabling detailed visualization of vascular anatomy, but is limited by ionizing radiation exposure and reduced accuracy in heavily calcified vessels [11–13]. Magnetic resonance angiography (MRA) provides high-resolution images without ionizing radiation and can be performed with or without gadolinium-based contrast agents; however, it is susceptible to artifacts from metal stents and carries a risk of nephrogenic systemic fibrosis in patients with renal dysfunction [14–16]. Nuclear imaging modalities, including positron emission tomography (PET) and single-photon emission computed tomography (SPECT), allow for molecular and functional imaging but are hindered by low spatial resolution, high cost, and limited ability to delineate vascular anatomy in detail [17–20]. Ultrasound (US) remains the mainstay of noninvasive PAD assessment due to its real-time imaging capability, safety, and accessibility, although it is operator-dependent and less sensitive for multilevel or distal stenoses [21–23]. Laser speckle contrast imaging (LSCI) offers label-free, high-temporal-resolution imaging of superficial blood flow but is restricted to very shallow imaging depths (< 2 mm) and cannot image deeper vessels relevant to PAD [24, 25].

**Table 1:** Comparison of imaging modalities for peripheral arterial disease (PAD)

Modality	Ionizing radiation	Contrast agent	Imaging depth	Spatial resolution	Temporal resolution	Cost and complexity	Limitation for PAD
Computed tomography angiography (CTA)	Yes	Iodine-based	Whole body	High (0.5–1 mm)	High (ms)	High	Limited accuracy in heavily calcified vessels
Magnetic resonance angiography (MRA)	No	Gadolinium-based (optional)	Whole body	High (0.5–1 mm)	Moderate (s)	High	Metal artifacts from stents; risk of NSF in renal disease
Positron emission tomography (PET)	Yes	Radiolabeled tracers	Whole body	Low (4–5 mm)	Low (s–min)	High	Cannot delineate arterial lumen or localize stenoses
Single-photon emission computed tomography (SPECT)	Yes	Radiolabeled tracers	Whole body	Low (~ 10 mm)	Low (s–min)	High	Cannot delineate the vascular anatomy in detail
Ultrasound (US)	No	Microbubbles (optional)	< 15 cm	High (0.1–3 mm)	High (ms–s)	Low	Unreliable flow measurements for multilevel stenosis
Laser speckle contrast imaging (LSCI)	No	Label-free	< 2 mm	Moderate (~ 10–100 μm)	High (ms–s)	Low	Cannot image deep vessels at all
Photoacoustic imaging (PAI)	No	Label-free	< 7 cm	High (1 μm–1 mm)	High (ms–s)	Moderate	Unable to provide whole body imaging depth

PAI has emerged as a promising non-ionizing modality that bridges optical and acoustic imaging by using pulsed laser light to generate acoustic waves from endogenous chromophores, such as hemoglobin, and detecting them with ultrasound transducers [26, 27]. This technique combines high spatial resolution (ranging from 1 μm to 1 mm, depending on system configuration) with functional and molecular imaging capabilities, such as mapping oxygen saturation and regional blood volume, offering insights into both macrovascular and microvascular pathophysiology [28–34]. PAI’s high temporal resolution enables dynamic imaging of hemodynamic responses, which is valuable for assessing perfusion and vascular reactivity [35–37]. Although current implementations of PAI are limited by imaging depth (approximately < 7 cm in human tissue), technological advancements continue to push these boundaries [38]. Importantly, PAI can operate label-free in many scenarios, reducing risks associated with contrast agents [39, 40]. This review critically examines recent developments in PAI technologies for PAD diagnosis, focusing on key advances in system design, preclinical validation, and emerging clinical applications that aim to address current diagnostic gaps and improve patient outcomes.

## Treatment Options

### Principles of PAI for PAD

PAI is a hybrid imaging modality that leverages the absorption of pulsed laser light by biological chromophores to generate broadband ultrasonic waves, which are then detected by ultrasonic transducers [28, 38, 41]. Specifically, when short laser pulses irradiate tissue, hemoglobin and other chromophores absorb optical energy, leading to transient thermoelastic expansion that produces broadband ultrasound waves [42, 43]. These waves propagate through tissue and are detected by acoustic transducers placed on the tissue surface. By analyzing the time-of-flight of the detected signals, the spatial distribution of absorbers, such as blood vessels and microvascular networks, can be reconstructed with high spatial fidelity [44]. This process combines the high optical absorption contrast of biological chromophores with the deep tissue penetration and spatial resolution inherent in ultrasound detection [31, 45]. PAI thus enables simultaneous visualization of vascular structures and functional parameters, such as total hemoglobin concentration and oxygen saturation, without relying on ionizing radiation or, in many cases, exogenous contrast agents [39, 42]. This feature is particularly advantageous in the context of PAD, where microvascular perfusion and oxygen delivery are often compromised [46, 47], and where repeated assessments may be required to monitor disease progression or therapeutic response [48, 49].

Recent developments in PAI have substantially expanded its potential clinical utility in PAD by enhancing temporal resolution, maximum imaging depth, and quantitative imaging capabilities [27]. Modern PAI systems can achieve sub-millimeter to micron-level spatial resolution, along with temporal resolutions in the millisecond range [28, 50]. These parameters are critical for capturing dynamic hemodynamic changes. For example, single-element detectors configured with ergodic relays and large-scale synthetic arrays have enabled rapid imaging [51–53], while advanced spectral unmixing algorithms facilitate quantification of oxygen saturation and regional blood volume with improved accuracy [36, 54]. Additionally, tunable excitation wavelengths allow for selective imaging of different tissue components, further enhancing the versatility of PAI [55–57]. These technological advances position PAI as a promising tool to assess both macrovascular and microvascular components of PAD pathophysiology, complementing standard anatomical imaging and providing insights into functional impairments that underlie clinical symptoms [58–60].

### Emerging PAI Techniques for PAD

#### High-Speed and Volumetric Imaging Systems

**Fig. 1:**
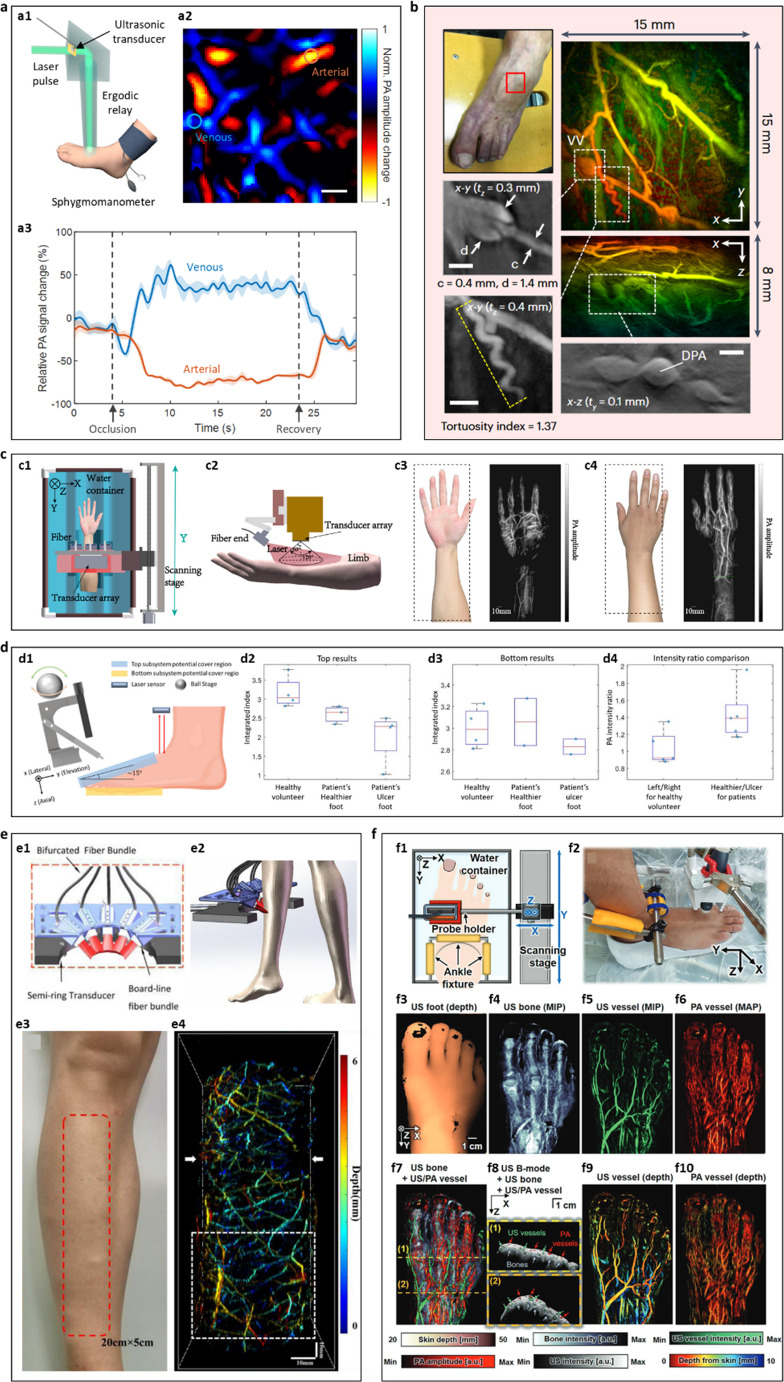
Emerging photoacoustic imaging (PAI) techniques for peripheral arterial disease (PAD). **a**, PAI with high-speed and volumetric imaging. (From Zhang Y et al. [63], with permission from Springer Nature). **a1**, Schematic illustration of the human foot imaging experiment using an ultrasonic transducer and ergodic relay. **a2**, Difference image showing the change in PA amplitude between baseline and vascular occlusion. **a3**, Relative PA signal changes in venous and arterial regions indicated by *blue* and *orange circles* in **a2**. *Shaded areas* represent standard deviation (*n* = 5). *Arrows* and *dashed lines* denote the start of occlusion and recovery. Scale bar, 1 mm. **b**, PAI with all-optical scanners and parallel detection. (Reproduced from Huynh NT et al. [64], https://www.nature.com/articles/s41551-024-01247-x; Creative Commons user license https://creativecommons.org/licenses/by/4.0/). Images acquired from a patient with suspected PAD. **Right**: x-y and x-z depth-encoded maximum intensity projections (MIPs). **Left**: expanded view of grayscale MIPs showing venous valve (**top left**) and corkscrew vessel (**bottom left**), with tortuosity index = 1.37 measured along the *yellow dashed line*. **Bottom right**: grayscale x-z MIP showing dorsalis pedis artery (DPA). **c**, PAI with large-scale synthetic aperture arrays. (Reproduced from Li S et al. [65], 10.1117/1.JBO.29.S1.S11519; Creative Commons user license https://creativecommons.org/licenses/by/4.0/). **c1**, Schematic diagram of the one-dimensional scanning process; red highlights the laser illumination area. Both the arm and the probe are immersed in water during imaging. **c2**, Schematic of fiber optic illumination system. **c3**, Photograph of the inner right palm and forearm, and corresponding 3D maximum amplitude projection (MAP) of the vasculature. **c4**, Photograph of the outer right palm and forearm, and corresponding MAP. **d**, Multi-view and dual-scan PAI. (Reprinted with permission from IEEE, from Huang C et al. [66]). **d1**, Schematic of top and bottom scanning setups, with *blue* and *yellow* highlighting potential scanning regions. A laser distance sensor is used for skin distance measurement, and the scanner is rotated via a ball stage in the axial-elevation plane to align with the foot. **d2**–**d3**, Quantitative comparisons of top (**d2**) and bottom (**d3**) scan features among healthy volunteers, healthy feet of patients, and ulcerated feet of patients. **d4**, *Box plots* of PA intensity ratios comparing left/right feet for healthy volunteers and healthy/ulcerated feet for patients; individual data points are shown. **e**, Dedicated portable PAI system for PAD (Reprinted with permission from IEEE, from et al. Chen T [67]). **e1**, Schematic of the optical and acoustic coupling module in a semi-ring transducer-based PAT system. **e2**, Schematic showing strip-type laser illumination projected onto human skin. **e3**, Photograph of the volunteer’s right lower leg with the *red dashed box* indicating the 20 × 5 cm imaging area. **e4**, Color-encoded depth-resolved 3D rendering of the vascular network. **f**, Multimodal PAI integrating ultrasound. (From Choi W et al. [68], with permission from the Radiological Society of North America). **f1**, Schematic of the PA/US foot scanner. **f2**, Photograph of the scanner setup on the patient’s foot. **f3**, 2D ultrasound image showing skin structure. **f4**, US MIP of bone. **f5**, US MIP of vasculature. **f6**, PA MAP of vasculature. **f7**, Overlay image combining **f4–f6**. **f8**, Cross-sectional images showing US B-mode (Brightness mode), US bone, US vessel, and PA vessel data. **f9**, Depth-encoded US vessel image. **f10**, Depth-encoded PA vessel image

Achieving high temporal and spatial resolution is essential for capturing dynamic vascular processes in the limbs, a key requirement for clinical translation of PAI. Traditional PAI systems often depend on mechanical scanning or array-based detection, which can be time-consuming and prone to motion artifacts [40, 61, 62]. To address these limitations, Zhang et al. developed a single-shot volumetric PAI system that uses an ergodic relay and a single-element ultrasonic transducer to record complete 3D images with each laser pulse [63]. As shown in Fig. 1(a1), this approach achieves real-time imaging at up to 1 kHz volume rates, enabling direct visualization of rapid blood flow dynamics during physiological challenges such as occlusion and reperfusion. The application of this technique to human foot imaging demonstrated clear separation of venous and arterial compartments (Fig. 1(a2)), with temporal PA signals revealing dynamic perfusion changes during occlusion and recovery (Fig. 1(a3)). This technique provides a transformative solution for real-time vascular monitoring without exogenous contrast agents or ionizing radiation, filling a critical gap in PAD diagnostics and offering potential for assessing exercise-induced ischemia and vascular reactivity.

#### All-Optical Scanners and Parallel Detection Systems

Complementing these advances in speed, Huynh et al. developed an all-optical 3D PAI scanner that leverages a Fabry–Perot (FP) polymer film sensor array with parallel detection [64]. This design, illustrated in Fig. 1(b), allows simultaneous acquisition of PA signals from multiple points, reducing scan time and improving sensitivity compared to conventional piezoelectric systems. By integrating a broadband pulsed laser with a tunable interrogation laser, the system captures high-fidelity signals with minimal crosstalk. Applied to the foot of a patient with suspected PAD, this system visualized detailed vascular features, including superficial and deeper vessels, corkscrew-shaped collaterals, and venous valves. The depth-encoded maximum intensity projections (MIPs) highlight both venous and arterial networks, while grayscale MIPs provide additional anatomical detail. Notably, the system’s rapid acquisition and high spatial resolution enhance clinical feasibility, offering a promising tool for real-time, noninvasive assessment of PAD.

#### Large-Scale Synthetic Aperture Arrays

To expand PAI’s field of view and resolution in peripheral vascular mapping, Li et al. introduced a large-scale synthetic matrix array system [65]. Using a one-dimensional transducer array on a motorized stage, the system synthesizes a high-density 2D detector matrix without the need for expensive 2D transducer arrays. The scanning geometry (Fig. 1(c1)) immerses both the limb and probe in water for consistent acoustic coupling, while fiber-optic illumination (Fig. 1(c2)) ensures uniform excitation across the scanned area. Applied to the palm and forearm, the system captured high-resolution 3D maximum amplitude projections (MAPs) that delineate both major and microvascular structures (Fig. 1(c3)–(c4)). This approach balances resolution and field of view, enabling detailed vascular visualization essential for assessing perfusion deficits, collateral formation, and other hemodynamic changes associated with PAD. Its mechanical scanning and cost-effectiveness position it well for point-of-care clinical use.

#### Multi-View and Dual-Scan Systems

Addressing anatomical complexity in the foot, Huang et al. developed a dual-scan PAI system that integrates dorsal (top) and plantar (bottom) views [66]. Traditional single-view PAI often suffers from incomplete coverage and acoustic shadowing in complex anatomical regions. The dual-scan design (Fig. 1(d1)) combines two scanning modules with laser distance sensors and a ball stage for precise alignment. Applied to both healthy volunteers and patients with diabetic foot ulcers, the system quantified vessel features including density, diameter, and tortuosity, revealing significant differences between healthy and diseased feet (Fig. 1(d2)–(d3)). PA intensity ratios (Fig. 1(d4)) further highlighted differences between contralateral feet, providing quantitative markers for disease severity. By reducing artifacts and enabling robust feature extraction, this system enhances clinical assessment of PAD and ulcer risk.

#### Dedicated Portable Systems

Portability and practicality are key for clinical adoption of PAI. Chen et al. developed a semi-ring array PAI system tailored for human peripheral vasculature [67]. Its design balances high resolution (~ 200 μm) with a large field of view (15 cm × 4 cm), completing scans in 50 s. As shown in Fig. 1(e1)–(e2), the system integrates a strip-type illumination system for uniform excitation, while the semi-ring array optimizes acoustic coupling and anatomical coverage. This configuration enables imaging in both standing and seated positions (Fig. 1(e3)), facilitating patient comfort and clinical workflow integration. Depth-resolved, color-encoded 3D vascular maps (Fig. 1(e4)) reveal sub-millimeter vessels, essential for evaluating microvascular health and collateralization. This portable system holds a strong promise for point-of-care PAD diagnostics.

#### Multimodal Imaging Approaches

Finally, Choi et al. demonstrated the integration of PAI with ultrasound (US) to provide both structural and functional information in PAD assessment [68]. Combining the high optical contrast of PAI with US’s anatomical detail, this multimodal system (Fig. 1(f1)–(f2)) enables simultaneous acquisition of soft tissue, bone, and vascular images. US images capture skin, bone, and vessel MIPs (Fig. 1(f3)–(f5)), while PAI provides functional maps of hemoglobin distribution (Fig. 1(f6)). Overlay and fusion images (Fig. 1(f7)–(f8)) integrate both modalities, allowing direct correlation of structural and functional data. Depth-encoded visualizations (Fig. 1(f9)–(f10)) further enhance interpretation of vascular networks and perfusion. This comprehensive approach improves diagnostic confidence and may guide treatment planning in clinical PAD management.

### Preclinical and Translational Research

Preclinical and volunteer studies have been instrumental in translating PAI from laboratory innovation to clinical application in PAD. In healthy volunteers, researchers have employed controlled ischemia–reperfusion models to validate the sensitivity of PAI to dynamic changes in perfusion and oxygenation. For example, Zhang et al. demonstrated that their single-shot volumetric PAI system could monitor rapid hemodynamic fluctuations in the human foot during arterial occlusion and subsequent reperfusion [63]. This approach highlighted the system’s potential for noninvasive, real-time assessment of vascular reactivity, which is a critical functional parameter in PAD evaluation. Likewise, Choi et al. applied exercise-induced occlusion protocols and quantified significant changes in oxygen saturation and total hemoglobin using PAI, providing direct evidence of the technique’s capacity to detect functionally relevant impairments in microvascular perfusion associated with PAD [68].

Building on these promising preclinical findings, translational research has advanced PAI into pilot studies involving patients with PAD. Karlas et al. utilized multispectral photoacoustic tomography to assess calf muscle oxygenation in patients with intermittent claudication [69]. Their study revealed significantly lower muscle oxygenation in PAD patients compared to healthy controls at both rest and post-exercise. These findings correlated with established clinical measures such as the ankle–brachial index and walking performance. Importantly, this work demonstrated the potential of PAI to provide functional insights that complement traditional anatomical imaging modalities. Further supporting this translation, Caranovic et al. validated a photoacoustic biomarker for PAD detection in a cohort of patients with intermittent claudication, achieving high sensitivity and specificity [70]. This study highlighted the technique’s promise for early disease detection and risk stratification, which are key steps in improving patient outcomes.

Collectively, these preclinical and translational investigations underscore the potential of PAI as a functional imaging modality that bridges the gap between microvascular physiology and clinical diagnosis in PAD. By enabling real-time, noninvasive assessment of tissue oxygenation and perfusion dynamics, PAI offers a valuable complement to existing anatomical imaging approaches, with the potential to refine diagnosis, guide treatment strategies, and monitor therapeutic efficacy.

### Translational Challenges and Future Directions

Despite notable progress in the development of PAI systems for PAD, several key challenges must be addressed before widespread clinical adoption can be realized. One fundamental limitation is imaging depth: while modern PAI systems can achieve imaging depths of up to 7 cm under optimal conditions, this is often inadequate for visualizing deeper-seated arteries in larger patients or in regions with substantial subcutaneous fat [38]. Consequently, PAI remains best suited for assessing superficial vessels and microvascular beds, serving as a valuable complement to established angiographic modalities such as CTA and MRA.

Another major barrier is the complexity and cost of current PAI systems. These platforms often rely on specialized lasers, sophisticated detector arrays, and advanced signal processing algorithms, which together increase the technical burden on healthcare providers [41]. Such complexity may necessitate additional staff training and infrastructure investment, posing practical challenges for routine deployment in vascular clinics. Moreover, while the quantitative capabilities of PAI, such as oxygen saturation mapping, are compelling, their accuracy depends on robust spectral unmixing algorithms [54]. These algorithms can be sensitive to factors like optical scattering, motion artifacts, and calibration inconsistencies, which can compromise measurement reliability and reproducibility across different devices and patient populations. Ensuring standardization and cross-platform validation of quantitative PAI metrics remains a critical step toward clinical translation.

In addition, regulatory pathways for PAI are not yet fully established. Specifically, standardized protocols for data acquisition, analysis, and reporting are needed to facilitate clinical adoption and ensure consistency with existing diagnostic workflows. Demonstrating the added clinical utility of PAI relative to established diagnostic modalities will be essential for regulatory approval and reimbursement. Large-scale clinical studies are therefore needed to validate PAI’s diagnostic and prognostic performance, and to define its role within existing PAD management frameworks [27, 71].

Nevertheless, the rapid technological progress in the field, including single-shot volumetric imaging [63], all-optical scanners with parallel detection [64], and multimodal integration with ultrasound [68], provides a strong foundation for overcoming these translational barriers. Continued collaboration among engineers, clinicians, and regulatory agencies will be essential to advance PAI from a promising research tool to a clinically viable technology. With its unique ability to provide real-time, noninvasive insights into microvascular health, PAI has the potential to transform PAD diagnosis and management, ultimately improving patient outcomes through enhanced functional assessment and more personalized treatment strategies [72, 73].

## Conclusions

In summary, emerging PAI technologies have demonstrated remarkable potential to transform the diagnosis and management of PAD. Innovations such as high-speed volumetric imaging, all-optical scanner arrays, large-scale synthetic apertures, and multimodal integration with ultrasound have collectively advanced the field, enabling real-time visualization of vascular structure and dynamic hemodynamics. These systems offer functional insights that complement conventional anatomical imaging, thereby supporting more precise and individualized therapeutic strategies.

Despite these promising developments, several challenges remain to be addressed to fully realize PAI’s clinical potential. Depth limitations currently confine PAI’s applicability to superficial vessels, while variability in system performance underscores the need for robust standardization and cross-platform validation. In addition, regulatory frameworks and clear clinical guidelines for integrating PAI into established diagnostic pathways are still evolving.

At the same time, promising opportunities lie ahead. The integration of artificial intelligence and machine learning could enhance image reconstruction, feature extraction, and automated interpretation, further bridging the gap between complex PAI datasets and clinical decision-making [29, 74–76]. The emergence of theranostic applications and the development of wearable or portable PAI systems also hold promise for continuous vascular health monitoring and personalized care [58, 77].

Ultimately, continued interdisciplinary collaboration among engineers, clinicians, and regulatory bodies will be vital to overcome these barriers and establish PAI as a standard tool for PAD diagnosis and management. As technological refinements continue and clinical validation expands, PAI is poised to play a pivotal role in the comprehensive assessment of PAD, offering noninvasive, real-time insights that can inform both preventative and interventional strategies.

## Key References

- Zhang Y, Hu P, Li L, Cao R, Khadria A, Maslov K, et al. Ultrafast longitudinal imaging of haemodynamics via single-shot volumetric photoacoustic tomography with a single-element detector. Nat Biomed Eng. 2024;8:712–25.
  - This study presents a single-shot volumetric PAI system enabling ultrafast imaging of dynamic hemodynamics in the human foot, demonstrating the potential of real-time monitoring of microvascular responses in PAD.
- Huynh NT, Zhang E, Francies O, Kuklis F, Allen T, Zhu J, et al. A fast all-optical 3D photoacoustic scanner for clinical vascular imaging. Nat Biomed Eng. 2025;9:638–55.
  - This work introduces an all-optical parallel-detection 3D PAI scanner that reduces acquisition times and enhances sensitivity, facilitating detailed vascular imaging in PAD patients.
- Park J, Choi S, Knieling F, Clingman B, Bohndiek S, Wang LV, et al. Clinical translation of photoacoustic imaging. Nat Rev Bioeng. 2025;3:193–212.
  - This comprehensive review outlines the fundamentals of PAI technology and highlights clinical systems, pilot studies, and patient trials in human organ systems. It also discusses technical and non-technical challenges and emphasizes the importance of standardization to accelerate clinical translation.

## Data Availability

No datasets were generated or analysed during the current study.
